# Pharmacokinetic and pharmacodynamic assessment of oral nicotinamide in the NEAT clinical trial for early Alzheimer’s disease

**DOI:** 10.1186/s13195-025-01693-y

**Published:** 2025-03-11

**Authors:** Gabriel L Ketron, Felix Grun, Joshua D Grill, Howard H Feldman, Robert A Rissman, Gregory J Brewer

**Affiliations:** 1 Department of Biomedical Engineering, University of California, Irvine, CA USA; 2 Department of Chemistry, University of California, Irvine, CA USA; 3 Institute for Memory Impairments and Neurological Disorders, University of California, Irvine, CA USA; 4 Center for Neurobiology of Learning and Memory, University of California, Irvine, CA USA; 5 Department of Neurosciences, University of California San Diego, La Jolla, CA USA; 6 Department of Neurobiology and Behavior, University of California, Irvine, CA USA; 7 Alzheimer’s Disease Cooperative Study, School of Medicine, University of California, La Jolla, CA USA

**Keywords:** Alzheimer disease clinical trial, Nicotinamide, Pharmacokinetics, Plasma, CSF, Methyl-nicotinamide, Ptau_231_

## Abstract

**Background:**

Nicotinamide, a form of B3 vitamin, is an NAD^+^ precursor that reduces pTau_231_ levels via histone deacetylase inhibition in murine models of Alzheimer’s disease (AD). A recent phase 2a randomized placebo-controlled trial tested high-dose oral nicotinamide for the treatment of early AD. While nicotinamide demonstrated good safety and tolerability, it did not significantly lower CSF pTau_231_, the primary biomarker endpoint of the study. Characterization of nicotinamide’s pharmacokinetics and metabolites in the blood and CSF is needed.

**Methods:**

In these post hoc, blinded analyses of plasma and CSF samples from the completed two-site placebo controlled randomized trial testing of 1500 mg PO BID oral nicotinamide, we used mass spectroscopy to measure nicotinamide and its inactive metabolite 1-methyl-nicotinamide in plasma at baseline, 6, and 12 months and in CSF at baseline and 12 months from 23 participants on drug and 24 on placebo.

**Results:**

Pharmacokinetic analysis found mean 12 month plasma nicotinamide increased > 130-fold to 52 μM while mean methyl-nicotinamide increased > 600-fold to 91 μM in individuals receiving nicotinamide compared to those receiving placebo, whose levels were unchanged from baseline. However, CSF nicotinamide was only measurable in 6 of the 19 available participants (32%) (mean increase of at least 147-fold to 18 μM). These CSF nicotinamide concentrations were 66% of their plasma levels, indicating good CNS bioavailability in only some participants. In contrast to CSF nicotinamide, more treated participants had higher CSF methyl-nicotinamide (*n* = 9, 43 μM), suggesting high-dosage nicotinamide was sufficient to pass the blood–brain barrier, but 13 of 19 were metabolically inactivated. Treatment favorably decreased mean pTau_231_ levels by 34% in those six participants with elevated CSF levels of nicotinamide, compared to 3% elevation in participants who did not have elevated CSF nicotinamide, and a 3% decrease for placebo. No such relationships were observed for total tau, pTau_181_, or amyloid beta biomarkers.

**Conclusions:**

Our findings suggest that oral administration markedly increased mean plasma nicotinamide levels, however CSF levels were below quantitation in a majority of participants and there was extensive metabolic inactivation to methyl-nicotinamide. Both the bioavailability and rapid metabolic methylation need to be addressed if nicotinamide is further developed as a potential intervention for AD.

**Trial registration:**

NCT03061474, last updated 2023–10-17. https://clinicaltrials.gov/study/NCT03061474.

**Supplementary Information:**

The online version contains supplementary material available at 10.1186/s13195-025-01693-y.

## Background

Nicotinamide serves as a co-enzyme precursor to NAD^+^ and NADH for cellular oxidation–reduction reactions. Inhibition of class III histone deacetylases (HDAC Sirtuins) affects numerous therapeutic pathways including inducing PI3k, MAPK/ERK42/44, cAMP and NAD^+^ production pathways, associated with neuronal survival, autophagy, neuroplasticity and lower oxidative stress [1, 2]. In mouse models of Alzheimer’s disease (AD), nicotinamide improved cognitive function while lowering Aβ and soluble p-tau isoforms [1, 2]. In these studies, nicotinamide in the drinking water reduced pTau_231_ in immunostained brain sections [1]. Sirt1 knockdown produced a similar effect suggesting nicotinamide inhibited the Sirt1 deacetylase. Nicotinamide also promotes NAD^+^ dependent sirtuin activity through increased NAD^+^ production.

Oral nicotinamide is absorbed in the small intestine and encounters some first pass metabolism in liver to inactive N-methyl-nicotinamide. Blood concentrations are also lowered by kidney filtration that may affect CNS bioavailability. For oral nicotinamide to be CNS active, it must penetrate the blood brain barrier without being inactivated [3]. Some reports indicate nicotinamide is transported across the blood brain barrier bidirectionally with non-saturable capacity [3], probably due to rapid phosphorylation. The phosphorylation of nicotinamide is mediated by nicotinamide phosphoribosyl transferase to make nicotinamide mononucleotide, which is rapidly converted by nicotinamide mononucleotide adenylyl transferase into NAD^+^ in the “two-step salvage pathway” [4]. NAD^+^ is reduced to NADH by dehydrogenases in the mitochondrial TCA cycle and oxidized back to NAD^+^ in the electron transport chain. Lastly, Sirt1, PARP and CD38 enzymes cut NAD^+^ by glycohydrolysis into nicotinamide and ADP-ribose [5]. Consumption and degradation pathways of nicotinamide in the gut microbiome, intestines, blood, and liver complicate drug delivery to the brain. Importantly, nicotinamide inhibition of sirtuins in cells is limited by its metabolism to 1-methyl-nicotinamide (methyl-nicotinamide hereafter) by nicotinamide N-methyl transferase [6]. These preclinical studies suggested a potential role for treating bioenergetic or acetylation deficits in AD and motivated a phase 2a proof-of-concept clinical trial.

The *Nicotinamide as an Early AD Treatment* (NEAT) study tested high-dose (1500 mg PO BID) oral nicotinamide in participants with Mild Cognitive Impairment (MCI) or mild dementia in whom a biomarker signature confirmed a diagnosis of AD. The primary outcome of the trial was change in CSF pTau_231_ [7]. Soluble varieties of phosphorylated tau (pTau) at specific epitopes such as threonine 231 (pTau_231_) and threonine 181 (pTau_181_) are established markers of AD pathology in brain and serve as early biomarkers in the CSF [8].

In the NEAT trial, nicotinamide treatment of participants with MCI and mild AD dementia did not significantly reduce CSF pTau_231_ compared to placebo [7]. There were non-significant trends toward reduced change in CSF pTau_181_ and total tau with nicotinamide treatment. In the current study, we undertook post hoc analysis of nicotinamide pharmacokinetics (PK), pharmacodynamic (PD) metabolic inactivation to methyl-nicotinamide, and the association between nicotinamide concentration change and pTau_231_ concentration changes using samples from the NEAT trial. These analyses were undertaken to address whether there were sufficient levels in plasma and CSF with this oral nicotinamide dosage in this study population over the 48-week treatment period. Furthermore, the PK/PD relationship of plasma and CSF levels of both nicotinamide and methyl metabolite were evaluated with change in pTau_231_ and pTau_181_ concentrations, and other Aβ biomarkers. To measure these metabolite concentrations, and the concentrations of various break-down products, we conducted a Liquid Chromatography Mass Spectrometry (LC/MS) study of all NEAT participant samples.

## Methods

### Participant samples

The NEAT trial participants were recruited at UCLA and UC Irvine and were required to meet eligibility criteria that included clinical criteria for mild cognitive impairment or mild dementia due to AD, confirmed by a CSF biomarker (CSF amyloid beta 42 (Aβ_42_) ≤ 200 pg/mL or a ratio of total tau to Aβ_42_ ≥ 0.39) [9]. Study participants were randomized to receive 1500 mg nicotinamide BID in a sustained released tablet expected to last between 5 to 7 h or matching placebo [7]. Blood was collected from all participants at 0, 6, and 12 months after overnight fasting and plasma rapidly obtained. CSF samples were collected at 0 and 12 months. Plasma and CSF samples were stored at −80 °C until further processing. All samples were included in analysis, with an interval from last dose of drug or placebo ranging from 6 to 432 h. This included *n* = 47 samples, of which four samples were significantly delayed from last dose by 108, 147, 340 and 432 h.. Among participants receiving nicotinamide, the median duration from last dose to PK sample was 13.8 ± 0.05 h. (S.E., *n* = 15) after exclusion of one sample with a 340 h. delay.

### Chemicals and reagents

Nicotinamide (N3376, NAM, > 98% purity), 1-methyl-nicotinamide chloride (SML0704, > 98% purity, HPLC grade), nicotinamide-β adenine dinucleotide hydrate (N7004, NAD^+^, > 96.5% purity, HPLC grade), and nicotinamide-2,4,5,6-d4 (762,970, Internal Standard, 98% purity) were purchased from Sigma-Aldrich (St. Louis, MO, USA. Acetonitrile (043166.K2, 99.8% purity) was from Alfa Aesar (Haverhill, MA, USA) and formic acid was acquired from Thermo Fisher Scientific (A117, Waltham, MA, USA). 18.2 mOhm ultrapure water used for dilutions was produced by a Thermo Scientific GenPure Pro UV/UF water purification system (Waltham, MA, USA). Human plasma and cerebrospinal fluid samples of 46 participants across three time points were provided as frozen 1 mL aliquots from the NEAT clinical trial [7].

### External and internal standard preparation

Stock solutions of 10 mM nicotinamide, methyl-nicotinamide chloride, nicotinamide adenine dinucleotide and stable isotope-labeled nicotinamide (nicotinamide-2,4,5,6-d) internal standard (IS) were prepared in 13.3% acetonitrile and 0.3% formic acid in 18 mOhm water to match sample solvents. Analyte stocks were further mixed and diluted to generate a serial threefold dilution series from 3.3 µM to 4.6 nM in 13.3% acetonitrile-0.3% formic acid for use in calibration curves. Each calibration dilution contained IS at a constant concentration of 1 μM. IS was also added to each patient sample to a final concentration of 0.5 μM. All solutions and QC samples were stored at −20 °C. The lower limit of quantification for each analyte was determined by finding the lowest calibration curve concentration that could both be distinguished with 95% confidence from blank readings and have a signal-to-noise ratio greater than 8 to 1 [10].

### Sample preparation

To prepare blinded de-identified plasma and CSF samples for mass spectrometry, 1 μL of IS solution (Internal Standard D4-nicotinamide diluted to 0.6 mM in 13.3% acetonitrile-0.3% formic acid) was mixed into 39 μL of sample. Protein was precipitated by adding 160 μL of −20 °C 99.7% acetonitrile-0.3% formic acid to the IS-sample mix. Samples were centrifuged for 20 min at 4 °C at 17,000 g. 40 μL of the supernatant was then diluted fivefold with 200 μL of 0.3% formic acid in an autosampler well plate (Waters186002643). All plasma and CSF analytes were determined simultaneously for groups of 4–20 participants from the 46 participants without knowledge of their treatment assignment. The results are listed by participant in the attached file (Supplementary Table 2).

### Mass spectrometer and analytical conditions

Samples were analyzed on a Waters Acquity UPLC Quattro Premier mass spectrometer in triple quadrupole electrospray positive (ESI +) mode with an Acquity CSH™ Fluoro-Phenyl (2.1 × 50 mm, 1.7 μm) column. For liquid chromatography separation, we modified the method of Chu et al. (2021). We used 0.3% formic acid in water as the mobile A phase, and 99.7% acetonitrile/0.3% formic acid as the mobile B phase. The injection volume was 10 μL in triplicate. The sample elution gradient was programmed as 0 – 0.5 min. in 100% A; 0.5–2.5 min, linear to 95% B; re-equilibrated from 2.5–4.5 min. in 100% A. The flow rate was 0.3 mL/min, and column temperature was 50 °C. Participant analytes below the limit of quantification were evaluated at that limit of 0.4 μM for nicotinamide and 0.1 μM for methyl-nicotinamide. Additional details of mass spectrometry are included in supplementary materials.

### Statistical methods

Microsoft Excel (Version 16.82, Microsoft, Redmond, WA, USA) was used for data management and statistical analysis. Treatment allocation was unblinded after all sample analyte levels were completed by mass spectrometry. We compared the randomized placebo and treatment groups for main analyte effects by two-tailed unpaired Student’s T Tests, with significance at p < 0.05, without adjustment for multiple comparisons in this exploratory analysis. We estimated the association between levels of nicotinamide and methyl-nicotinamide with changes in CSF p-tau_231_, CSF pTau_181_, total Tau, CSF Aß_42_, CSF Aβ_40_, and Aβ_42/40_ ratio via linear regression. We also measured the within-participant association between analytes. Treatment group-level comparisons were further divided into an optimized difference between two groups with an elevated plasma or CSF nicotinamide or methyl-nicotinamide (> 2.1 μM) and drug unelevated participants (≤ 2.1 μM). We did not include other co-variates of sex, race, education, or ApoE status due to the small sample size. All bar graphs show mean and standard errors.

## Results

### Pharmacokinetics and dynamic drug metabolism: plasma

**Fig. 1:**
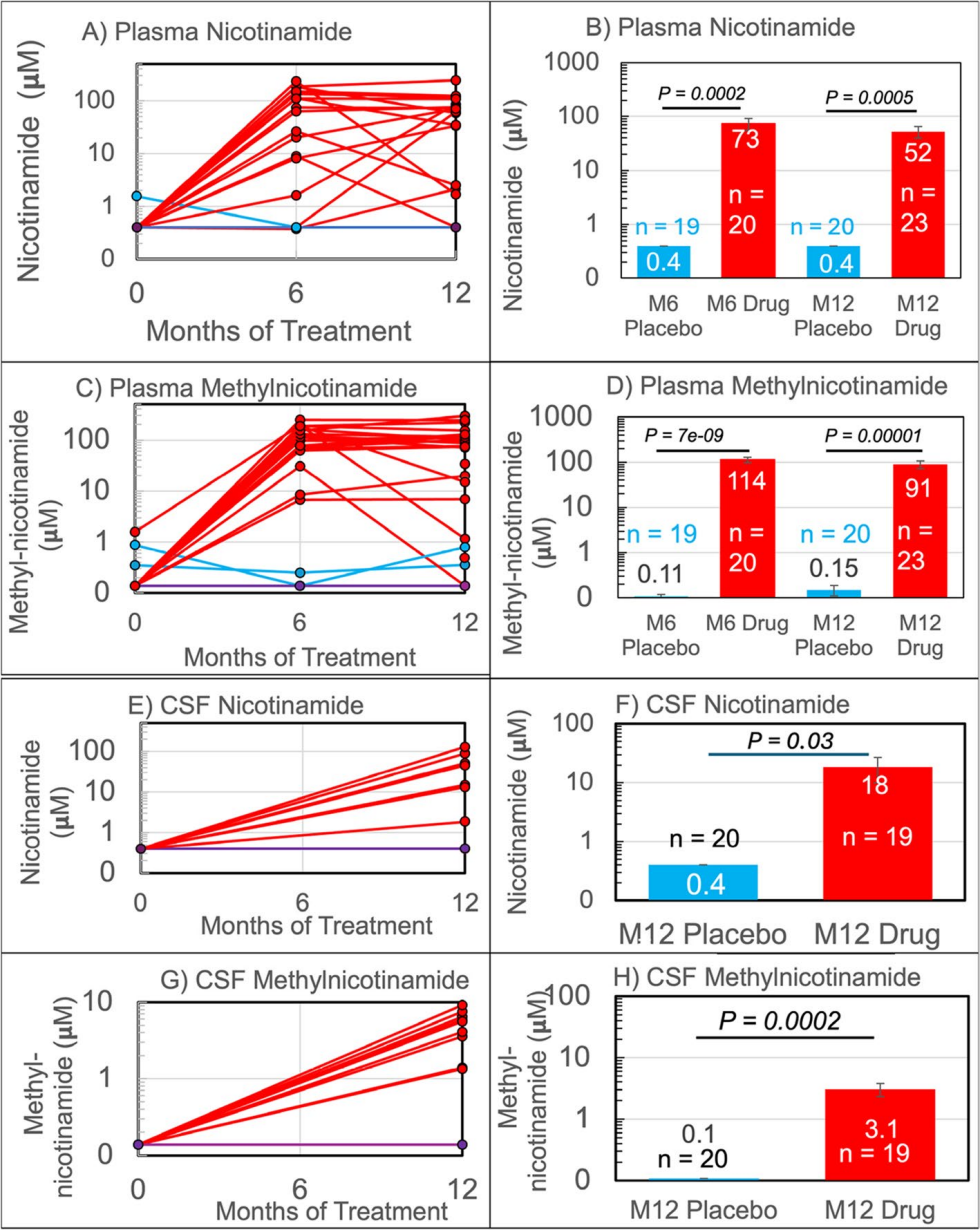
Pharmacokinetics of nicotinamide and methyl-nicotinamide in patient plasma and CSF. Red = Treatment, Blue = Placebo. **A** Each patient’s longitudinal changes in plasma nicotinamide concentration over time. Many data overlap at the minimum of quantification, 0.4 μM. **B** Average concentration of plasma nicotinamide at 6 months (M6) and 12 months (M12) for drug and placebo recipients. **C** Each patient’s changes in plasma methyl-nicotinamide concentration over time. **D** Average concentration of plasma methyl-nicotinamide at 6 and 12 months for drug and placebo recipients. **E** Each patient’s changes in CSF nicotinamide concentration with treatment. Many data overlap at the minimum of quantification, 0.1 μM. **F** Average concentration of CSF Nicotinamide at 12 months for drug and placebo recipients. **G** Each patient’s longitudinal changes in CSF methyl-nicotinamide concentration. **H** Average concentration of CSF methyl-nicotinamide at 12 months for drug and placebo recipients. Most participants remain at unelevated levels

The randomized study population included 47 participants with MCI or AD confirmed by CSF biomarkers of either Aβ_42_ ≤ 200 pg/ml or a ratio of total tau to Aβ_42_ > 39. Of these, 43 provided blood samples post baseline: 23 participants randomized to nicotinamide and 20 to placebo [7]. These participants were compared at three time points for plasma and two times for CSF samples (*n* = 39) utilizing liquid chromatography mass spectrometry analysis for nicotinamide and N-methyl-nicotinamide concentrations. Figure 1 shows the time-course for plasma analytes at 0, 6, and 12 months. At baseline before treatment (time 0), plasma nicotinamide concentration was below the limit of quantification of 0.4 μM in placebo and drug-treated participants. After 12 months, the placebo group was still at 0.4 μM. Baseline plasma metabolic inactivation product methyl-nicotinamide was above the 0.1 μM limit of quantification at 0.14 μM. As seen in Fig. 1A, plasma samples for all of the placebo-treated and a few of the nicotinamide-treated participants remained at baseline levels over the duration of the study. Among the nicotinamide-treated participants (Fig. 1A-B), mean plasma nicotinamide increased at least 183-fold to 73 μM at 6 months. These values included 15 values above baseline threshold at 6 months of the 20 participants receiving nicotinamide. Plasma nicotinamide rose at least 131-fold to 52 μM at 12 months with 17 of the 23 participants above their baseline levels. Incorporating the time of last dose to time of blood sample collection, we observed plasma nicotinamide elevated above baseline in blood samples from nicotinamide-treated participants collected between 6 and 16 h. delay without time dependence. In one participant with a 27 h. delay, plasma nicotinamide was measurable at a low value of 2 μM. In another with a 340 h. delay, nicotinamide was at or below our 0.4 μM threshold. Three baseline participants did not provide samples at 6 months. One participant who was above threshold at 6 months dropped below threshold at 12 months. Overall, metabolic inactivation product methyl-nicotinamide was elevated in plasma over 600-fold to a mean of 114 and 91 μM (6 and 12 months) in the 23 participants in the treatment group (Fig. 1 C-D), including the sample collected at 340 h. after the last dose.

### Pharmacokinetics and dynamic drug metabolism: CSF

CSF levels of nicotinamide and methyl-nicotinamide were analyzed to assess brain penetration. Prior to treatment, CSF concentrations were at or below the limit of quantification in all participants (0.4 𝝻M nicotinamide, and 0.1 𝝻M methyl-nicotinamide). Those on placebo were also at these limits of 0.4 𝝻M nicotinamide, and 0.1 𝝻M methyl- nicotinamide at 12 months. In Figure 1E-F, mean CSF nicotinamide at 12 months increased at least 45-fold to 18 μM with drug treatment. In Figure 1G-H, CSF mean methyl-nicotinamide at 12 months increased at least 31-fold to 3.1 μM.

### Drug levels elevated and unelevated in participant plasma and CSF

**Fig. 2:**
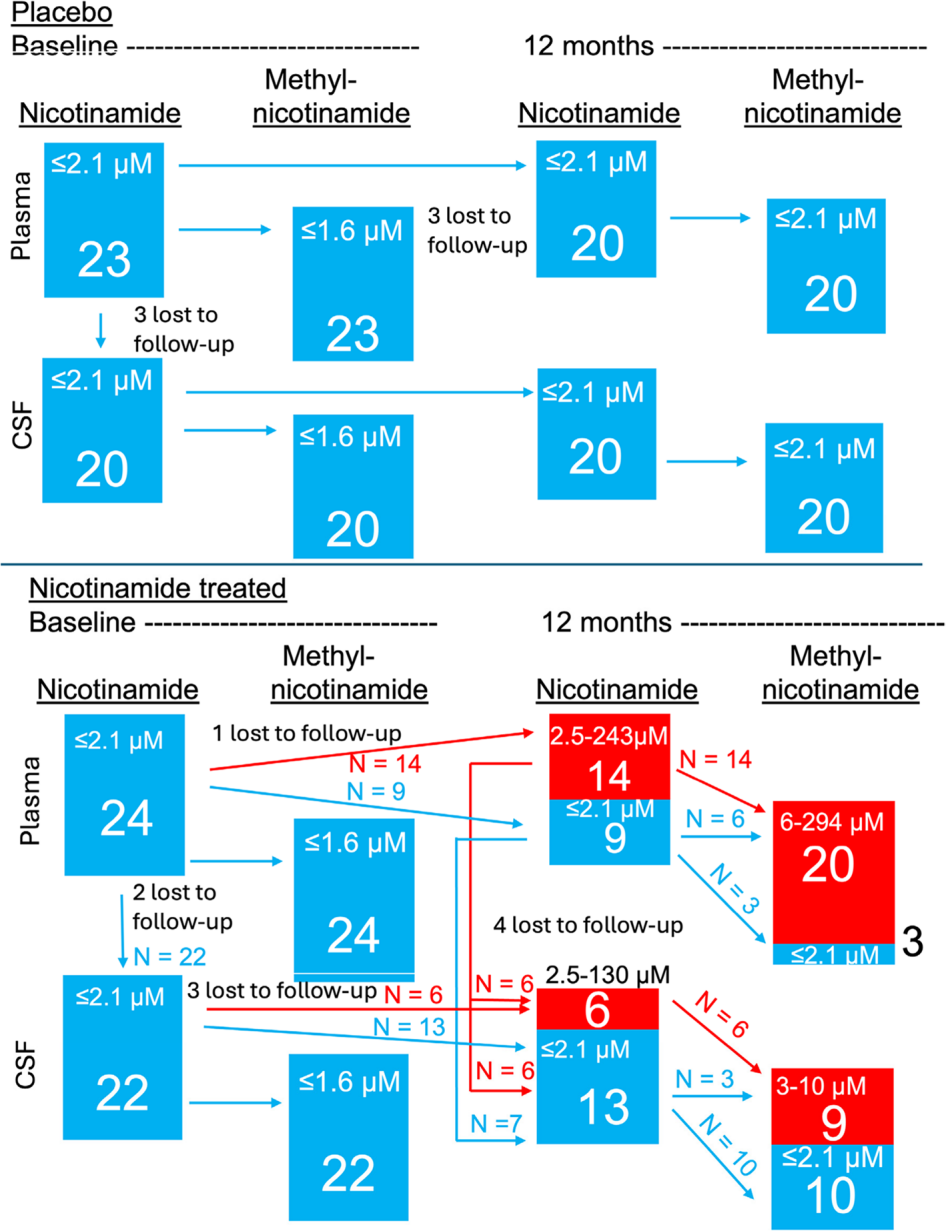
Distribution of participants by levels of nicotinamide and methyl-nicotinamide after 12 months of treatment. Note high numbers of treated participants with low plasma and CSF nicotinamide (blue bars). Note higher numbers of participants with high methyl-nicotinamide in both plasma and CSF of treated participants (red bars)

Figure 2 provides more detail for individual sub-group trajectories, accounting for study dropouts and participants whose analytes remained low. The constant baseline measures for the placebo group contrast with elevated measures of analytes in the treatment group. However, only 61% (*n* = 14 of 23) of the participants treated with nicotinamide for 12 months produced elevated plasma nicotinamide (*n* = 9 plasma nicotinamide levels remained unelevated). A possible explanation was suggested by detection of metabolic product methyl-nicotinamide in the plasma in 87% (*n* = 20 of 23) of the treated participants. The CSF results were more polarized. After 12 months of treatment, CSF nicotinamide above baseline levels was detected in only 32% (*n* = 6 of 19) of treated participants. Half of these CSF samples with elevated nicotinamide came from participants with unelevated plasma nicotinamide, supporting the idea that methyl-inactivation occurred primarily in the plasma, CSF methyl-nicotinamide was seen in 47% (*n* = 9 of 19) of treated participants. Therefore, unelevated levels of these analytes were found in 53–63% of participants. Furthermore 67% (*n* = 6 of 9) of these treated participants with unelevated plasma levels of nicotinamide showed elevated levels of methyl-nicotinamide in plasma. In CSF, *n* = 3 of 13 or 23% of participants with unelevated levels of nicotinamide showed elevated levels of methyl-nicotinamide (see Fig. 2). These data of plasma nicotinamide in the treated participants.

**Fig. 3:**
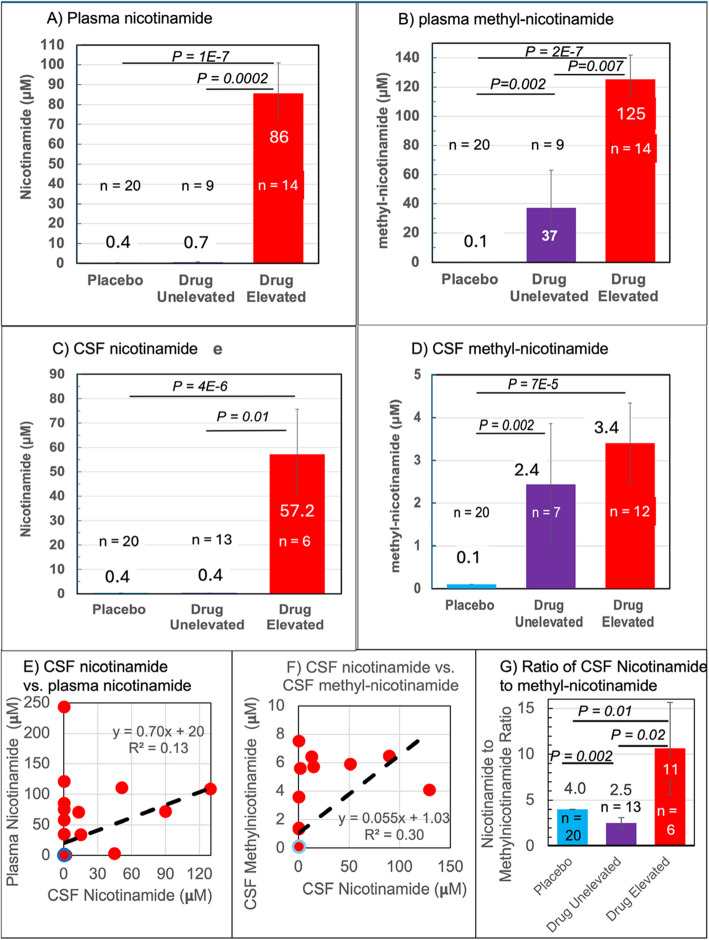
Segregation of 12 month plasma and CSF nicotinamide and methyl-nicotinamide into drug unelevated (≤ 2.1 μM) and drug elevated (> 2.1 μM) classes. **A** Plasma nicotinamide averages based on these classifications. **B** Plasma methyl-nicotinamide averages based on plasma nicotinamide elevated participants. **C** CSF nicotinamide averages for the participants with elevated CSF nicotinamide. **D** CSF methyl-nicotinamide averages based on CSF nicotinamide elevated participants. **E** Correlation of CSF nicotinamide concentration after 12 months of treatment to plasma nicotinamide among drug elevated participants (blue circle is level of non-elevated participants) A poor linear fit is shown (black dashed line). **F** Correlation of CSF nicotinamide concentration with CSF methyl-nicotinamide. **G** Ratio of CSF nicotinamide to CSF methyl-nicotinamide depend on whether nicotinamide was elevated in the plasma

The data in Figs. 1 and 2 indicate increased nicotinamide levels in some participants treated with nicotinamide while others did not demonstrate increased plasma and CSF levels. Treated participants with plasma nicotinamide at baseline levels of 0.4 μM up to 2.1 μM were designated drug unelevated; those above 2.1 μM as drug elevated. Figure 3A shows that this subgrouping of treated participants raised the average plasma nicotinamide from a mean 52 to 86 μM (at least 215-fold above baseline, 86/0.4). Surprisingly, Fig. 3B shows metabolic inactivation product methyl-nicotinamide increased to a mean 125 μM in the drug elevated group (at least an outsized 1250-fold above baseline and placebo; 1.5-fold above mean plasma nicotinamide). Also, even in the 9 participants with unelevated plasma nicotinamide (< 0.4 μM), methyl-nicotinamide levels averaged 37 μM. This suggests that the reason for treatment resulting in unelevated plasma levels for these 9 participants of 23 (39%) is that the drug was entirely methylated or entirely adsorbed into tissue.

Using these thresholds, Fig. 3C shows that the same subgroup analysis for CSF nicotinamide elevated participants which increased the mean CSF nicotinamide from 18 to 57 μM. In Fig. 3D, the participants with unelevated CSF nicotinamide of significantly increased their CSF methyl-nicotinamide from baseline of ≤ 0.1 μM up to a mean 2.4 μM (6% of plasma 37 μM). In participants with elevated CSF nicotinamide (6 of 19 participants receiving nicotinamide) CSF methyl-nicotinamide rose to a mean 3.4 μM (3% of 125 μM drug elevated plasma methyl-nicotinamide). Compared to the 1.5-fold elevation of plasma methyl-nicotinamide above plasma nicotinamide (125/86), the CSF methyl-nicotinamide was only 6% of CSF nicotinamide (3.4/57). If transport of plasma nicotinamide to the CSF were constant in all participants, then their relative concentrations would be linearly correlated. In drug elevated participants, Fig. 3E shows a poor correlation between plasma nicotinamide and CSF nicotinamide, suggesting factors other than blood brain barrier transport were involved. Using the same logic, Fig. 3F shows a poor correlation between CSF nicotinamide and CSF methyl-nicotinamide, suggesting that the level of N-methyl transferase activity in the CSF does not determine nicotinamide levels. Groupwise, in contrast to plasma, Fig. 3G shows ratios of CSF nicotinamide to methyl-nicotinamide that differ widely between drug unelevated and elevated participants. These subgroup difference suggest higher methylation contributed to the CSF nicotinamide in the unelevated group and lower methylation (11-fold higher ratio) allowed elevation of CSF nicotinamide in treated participants. The CSF to plasma ratio of nicotinamide was 66% (57/86) indicative of good drug penetration to the CNS among drug elevated participants. The ratio of elevated plasma methyl-nicotinamide to CSF methyl-nicotinamide was 37 (125/3.4), suggesting greater metabolism in the plasma than the CSF. Four participants assigned to nicotinamide did not demonstrate measurable plasma levels at 6 months (< 0.4 μM); seven did not demonstrate measurable plasma levels at 12 months. However, methyl-nicotinamide increased in 6 of these 7 nicotinamide unelevated participants to a mean of 12.7 μM methyl-nicotinamide (at least 127-fold above baseline). These measurements indicate that nicotinamide was elevated in the blood of only 61% of those treated (14/23), but 96% of treated participants (22/23) displayed elevated metabolic inactivation product methyl-nicotinamide. This suggests that nicotinamide reached the plasma in 97% of the treated participants, but was quickly metabolized to methyl-nicotinamide to the extent that the parent nicotinamide was no longer detectable. Induction of methyl transferase could explain why some participants’ nictotinamide were elevated at 6 months and then declined.

### Correlations of plasma and CSF nicotinamide with CSF pTau_231_

**Fig. 4:**
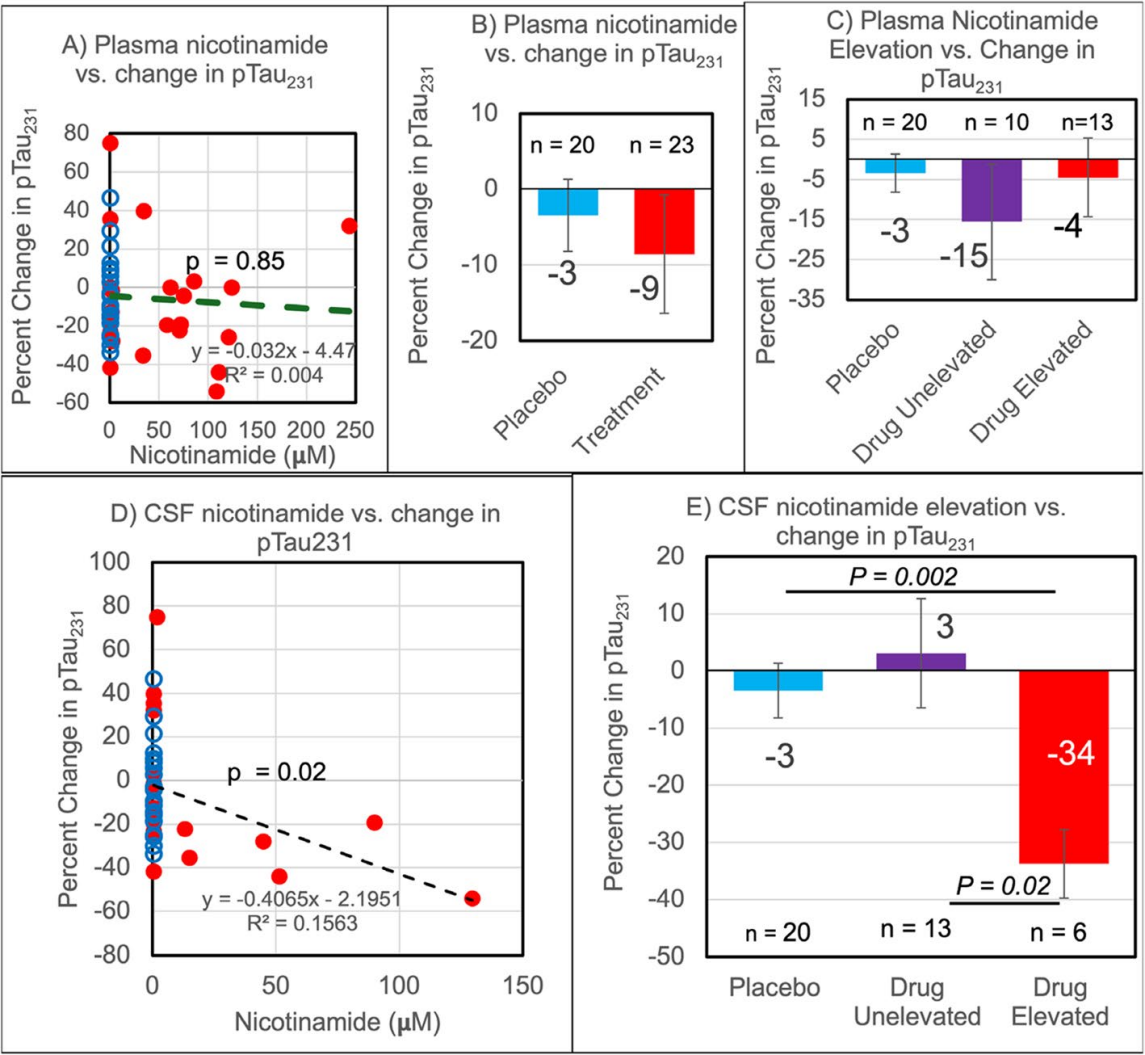
Correlation of plasma and CSF nicotinamide to reductions in pTau_231_ at 12 months. **A** Changes in CSF pTau_231_ concentration as a function of plasma nicotinamide concentration after 12 months of treatment (red) or placebo (blue). An insignificant linear fit of treated and placebo participants is shown (black dashed line). **B** Group-wise, percent change in CSF pTau_231_ shows no significant difference between all plasma placebo and all plasma treatment participants. **C** Treated participants with at least twofold elevated plasma nicotinamide did not show significant reductions in pTau_231_ concentration when compared to treated participants with unelevated plasma nicotinamide or the placebo group. **D** A weakly significant linear fit of change in plasma pTau_231_ concentration as a function of CSF nicotinamide concentration after 12 months of treatment and placebo (black dashed line). **E** Treated participants with at least tenfold elevated CSF nicotinamide saw greater reductions in pTau_231_ concentration when compared to treated participants with unelevated CSF nicotinamide or the placebo group

We explored correlations for participant-level plasma and CSF nicotinamide levels with AD biomarker outcomes. Figure 4A shows no correlation of pTau_231_ concentration with 12-month plasma nicotinamide concentration. Similar analyses for pTau_231_ failed to correlate with plasma methyl-nicotinamide, and CSF methyl-nicotinamide (not shown). Figure 4B compares placebo and treatment by group; there was no significant difference between the two groups. Two pharmacokinetically distinct groupings in the treated patient CSF nicotinamide drive this difference. Subgrouping into elevated and non-elevated plasma nicotinamide did not yield a significant association with change in pTau_231_ (Fig. 4C). However, as seen in Fig. 4D, with increasing CSF nicotinamide in drug treated participants, pTau_231_ favorably decreased. In the subgroups based on CSF nicotinamide analysis of Fig. 4E, pTau_231_ at 12 months decreased 34% from baseline in the six participants with elevated CSF nicotinamide compared to a 3% increase the CSF unelevated group or a 3% decrease in placebo groups. Thus, participants with elevated nicotinamide in their CSF may have demonstrated a favorable decreased pTau_231_.

### Other CSF biomarkers

We did not observe similar relationships of change in CSF pTau_181_, total tau or various CSF biomarkers for Aβ (Table 1). Plasma and CSF measures obtained simultaneously were below our limits of detection for nicotinic acid, nicotinamide mononucleotide and NAD^+^.

**Table 1:** Nonsignificant CSF biomarker based on non elevated CSF nicotinamide levels (≤ 2.1 μM) and elevated nicotinamade (≥ 2.1 μM) at 12 months (mean ± S.E.)

	Placebo	CSF Nicotinamide Non elevated	CSF Nicotinamide Elevated	All Treated
	*n* = 20	*n* = 13	*n* = 6	*n* = 19
Change in total tau (pg/mL)	100 ± 66	1.6 ± 70	−35 ± 84	−8.6 ± 54
Change in pTau_181_ (pg/mL)	10 ± 9.3	0.1 ± 8.5	1.1 ± 14	0.4 ± 7
Change in Aβ40 (pg/mL)	−1961 ± 1002	−2530 ± 754	−1823 ± 1094	−2307 ± 593
Change in Aβ42 (pg/mL)	−103 ± 72	−151 ± 52	−75 ± 44	−127 ± 38
Change in Aβ42/40 (%)	−4.9 ± 1.5	−4.5 ± 1.8	−2.1 ± 4	−3.7 ± 1.7
Change in total tau/ Aβ42(%)	44 ± 7	43 ± 8	35 ± 15	41 ± 7

## Discussion

Following 12 months of oral administration of nicotinamide to MCI and mild-AD participants enrolled in the NEAT trial [7], we evaluated the plasma and CSF levels of nicotinamide and methylated metabolic inactivation. Oral nicotinamide successfully passed into the blood stream, as indicated by at least 215-fold increases in mean nicotinamide to 86 μM (86/ <0.4) measured in 61% of the drug elevated recipients’ blood (14/23) compared to their baseline levels before drug. For participants with measurable nicotinamide in the plasma (average 86 μM), levels in the CSF were substantial at an average 57 μM, or 66% (57/86) drug penetration into the CSF, if not metabolized to methyl-nicotinamide. However, there was greater than a 1250-fold increase in average metabolism of nicotinamide to methyl-nicotinamide in drug elevated recipient’s plasma (125/ <0.1) compared to smaller increases in the CSF, suggesting a high rate of drug metabolism in the plasma before reaching the CNS. Intriguingly, among AD biomarkers, only CSF pTau_231_ was favorably impacted by a 34% reduction in treated participants with elevated nicotinamide, compared to a 3% reduction in placebo at 12 months.

Elevated nicotinamide was measurable in CSF in only 32% of the drug recipients (6/19) with a mean increase of at least 142-fold from baseline (57/ ≤ 0.4)). The remaining 68% did not achieve measurable CSF levels. Three of the treated participants (13%) had an increase only in CSF methyl-nicotinamide implying that most nicotinamide was adsorbed or fully methylated before reaching the CNS. Ten of the 19 treated participants (52%) showed no evidence of nicotinamide or methyl-nicotinamide in the brain suggesting either rapid uptake and conversion to NAD^+^ [3, 4], or significant methylation in the blood [11]. The possibility of nicotinamide not passing the blood brain barrier seems less likely given prior reporting [3] and our measures of substantial CSF levels of nicotinamide in a subset of participants.

We compared our baseline human blood and CSF measurements of nicotinamide, methyl-nicotinamide, and NAD^+^ to other mass spectrometry techniques in Table 2. In general, our nicotinamide and plasma methyl-nicotinamide baseline measurements are similar to other reports. Our baseline and placebo measurements agree with Chu et al. [12], and Ibrahim et al. [13] for an obese presumably cognitively unimpaired population. Baseline high concentrations of plasma 30–40 μM nicotinamide were reported in Clement et al. [14] for a normal aging, non-demented middle-age population and in Seyedsadjadi et al. [15] for a middle-age population, much higher than our 0.4 μM, perhaps due to some methodological differences in sample collection or processing. For participants without AD, Chu et al. [12], and Galla et al. [16] measured the same levels of plasma methyl-nicotinamide as we did, while those of Clement et al. [14] and Seyedsadjadi et al. [15] were 60–80% lower. Our methods failed to measure plasma NAD^+^ above 1.2 nM, but Clement et al. [14] and Seyedsadjadi et al. [15] found NAD^+^ in the ten-fold lower. Galla et al. [16] reported CSF methyl-nicotinamide at around 39 nM, lower than our 0.1 μM limit of detection Our NAD^+^ and CSF methyl-nicotinamide limits of detection were too high to corroborate other papers’ findings. However, across each study, sample preparation and mass spec machines were different, which limits strict comparisons.

**Table 2:** Comparison of our analyte concentrations to other publications (all µM ± S.D.). N/A is not available

Reference > Analyte	This work Baseline (*n* = 47)	Chu et al. [12] (*n* = 659)	Ibrahim et al. [13] (*n* = 54)	Clement et al. [14] (*n* = 10)	Seyedsadjadi et al. [15] (*n* = 100)	Galla et al. [16] (*n* = 10)
Plasma Nicotinamide	0.5 ± 0.1	0.9 ± 0.02	1.2 ± 0.14	38 ± 11	32 ± 11	N/A
Plasma methyl-nicotinamide	0.14 ± 0.03	0.1 ± 0.002	N/A	0.05 ± 0.01	0.033 ± 0.012	0.185 ± 0.116
Plasma NAD^+^	< 1.2	N/A	N/A	0.008 ± 0.02	0.023 ± 0.012	N/A
CSF Nicotinamide	< 0.4	N/A	N/A	N/A	N/A	N/A
CSF methyl-nicotinamide	< 0.1	N/A	N/A	N/A	N/A	0.039 ± 0.025

### Metabolism of nicotinamide by nicotinamide N-methyl transferase

Many nicotinamide treated participants with low CSF nicotinamide also had high levels of CSF methyl-nicotinamide, suggesting that nicotinamide had crossed the blood brain barrier, but was methylated to lower effective concentrations. Since nicotinamide treated participants showed significant increases in plasma methyl-nicotinamide, low nicotinamide levels may similarly reflect consumption of available nicotinamide. The finding from three treated participants with unelevated plasma nicotinamide at 12 months, but quantifiable levels at 6 months, suggests there was increased induction of nicotinamide N-methyl transferase, the enzyme methylating nicotinamide [17] that prevented CNS accumulation of nicotinamide. Unusually high levels of nicotinamide N-methyl transferase have been associated with Parkinson’s and Alzheimer’s diseases [18]. This could be a stress response to underlying disease pathology, or a response to excess nicotinamide in an environment that lowers enzyme activity, such as an oxidative redox state, or lower S-adenosyl methionine as methyl donor [19]. Methylation activity not only consumes nicotinamide, but also lowers Sirtuin3 activity by lowering substrate NAD^+^ levels [2].

Some of the discrepancy in biomarker outcomes between the preclinical mouse study and the NEAT human trial may emerge from differences in nicotinamide N-methyltransferase (NNMT) levels between humans and mice. Human NNMT activity was 51 nM methyl-nicotinamide formed per hour per mg of liver protein [17]. Mouse NNMT was one-third of that at 16 nM [20]. On top of this, participants with AD have 7.5 times more NNMT protein in the medial temporal lobe than healthy participants [17]. When Green’s 3xTg-AD young mice were tested [1], they may have been able to take advantage of nicotinamide consumption through rapid accumulation, whereas the human NEAT study participants were older. They may have inactivated nicotinamide more rapidly, resulting in less accumulation of CSF nicotinamide.

Our findings suggest that people with AD who have lower CSF nicotinamide also have higher plasma nicotinamide N-methyl transferase, thereby metabolizing nicotinamide before a measurable change in Sirt-mediated pTau_231_ reduction could take place, and simultaneously promoting additional Sirt3 acetylation. Methyl-nicotinamide itself binds to and stabilizes Sirt1 [21], possibly blocking inhibition by nicotinamide. Measurement of plasma and CSF nicotinamide N-methyl transferase levels could confirm that these people clear nicotinamide much faster due to methylation or another mechanism. Combining nicotinamide with a reversable nicotinamide N-methyl transferase inhibitor, such as oral 6-methyloxy-nicotinamide (JBSNF-000088) to prevent methylation in the bloodstream may be a solution to increase CNS nicotinamide bioavailability [22]. If used in tandem, JBSNF-000088 may compete with oral nicotinamide (IC_50_ of 1.8 μM) and allow for sufficient CNS accumulation even among participants with high levels of nicotinamide N-methyl transferase.

### Limitations

The greatest limitation of this study was the small sample size, especially as there were only six participants who achieved elevated CSF nicotinamide. However, enriching the group of people to be considered for treatment with nicotinamide to those with low plasma nicotinamide N-methyl transferase could help select likely responders more efficiently. This direction could begin a more precision medicine based approach to testing nicotinamide for AD. Our method of adjusting for matrix effects that suppress recovery involved inclusion of a deuterated form of nicotinamide at 15 μM in each sample. This single concentration may have affected the accuracy of measurements far from this value. However, our accuracy from plasma spiked with analyte measured at 116% of expected was good and other measures should be similar since 94% (15/16) of the measurable nicotinamide levels were within one log of the internal standard. The associated issue of methylation may have precluded correlations between CNS nicotinamide and changes in their biomarker scores on either pTau_181_, total tau, or Aβ. We note that our findings were similar to the Green et al. [1] mouse study that reported oral nicotinamide treatment failed to change pTau_181_, total tau, or Aβ, but did see a reduction in pTau_231_ concentration. The similarity in findings in a select group seem to support the specificity of nicotinamide targeting pTau_231_, though the mechanism of such specificity remains uncertain. Three postulated theories in the mouse study included Sirtuin 1 inhibition promoting pTau_231_ degradation, ubiquitination inhibition, or another mono-ubiquitination related pathway. Sirtuin 1 knockout produced similar effects as nicotinamide in the mouse study and was associated with an increase in acetylated α-tubulin [1]. The small number of high CSF nicotinamide participants also raise the alternative possibility that measured reductions in pTau_231_ are not representative of CSF nicotinamide treatment. This result could also be due to nicotinamide not acting as a histone deacetylase inhibitor as hypothesized by the NEAT trial. We were limited by not having samples such as red blood cells to examine the intracellular nicotinamide metabolome. A more direct measure of target engagement would be a rise in intracellular NAD^+^ from nicotinamide dosing, as has been demonstrated for oral nicotinamide riboside and nicotinamide mononucleotide [23, 24]. These energy precursors are in current clinical trials for AD [Hanoglu, NCT04044131; Haukeland University Hospital, NCT05698771; Abinopharm Inc, NCT04823260]. The development of in vivo ^31^P Magnetic Resonance Spectroscopy including assessment of NAD metabolic state and redox with high field MR holds some promise to more fully evaluate this important energy metabolome [25].

### Future directions

Our findings suggest that higher CSF concentrations of nicotinamide reduced pTau_231_ concentration in a limited subset of treated participants, perhaps because metabolism of drug prevented this observation in other treated participants. Given the high levels of methylated nicotinamide in treated participants, plasma methyltransferase assays may reveal which participants have lower methyl-transferase activity to screen for a population most likely to respond to treatment.

Future trials of nicotinamide, if undertaken, might also consider co-treatment with inhibitors of nicotinamide N-methyl transferase. Overexpression of this enzyme has been linked to cancers, metabolic disorders and neurodegenerative diseases like AD, Parkinson’s Disease and Huntington’s Disease [26], but whether this elevated expression is pathologic or protective is unclear. A variety of inhibitors of nicotinamide N-methyl transferase have been developed as cancer treatments [27]. Inhibitors fall into four categories based on their method of inhibition; S-adenosyl-l-methionine (SAM) binding site inhibitors, nicotinamide binding site inhibitors, bisubstrate inhibitors which block both, and covalent inhibitors which bond to nicotinamide N-methyl transferase. SAM binders are toxic in animal models since SAM is a requisite substrate for the enzyme [28]. Bisubstrate inhibitors bind very well to nicotinamide N-methyl transferase with Ki values as low as 0.5 nM but did not inhibit well in vivo. Covalent inhibitors were not very effective even at very high concentrations. Nicotinamide competitive inhibitors are not the most potent inhibitors but may provide effective and safe reductions in methylation. JBSNF-000088 was found to reduce methyl-nicotinamide in mouse plasma by 50% but reduced body weight. Another inhibitor, 5-amino-1-methylquinoline was found to improve muscle regeneration in aged mouse models [26]. In our investigation only 30% of the treatment group saw CSF nicotinamide accumulation after 12 months, but methyl-nicotinamide significantly increased in 45% of participants. Furthermore, 30% of participants appeared to completely methylate all nicotinamide in the blood by the 12-month blood draw, preventing nicotinamide from reaching the CSF. Inhibition of nicotinamide N-methyltransferase with JBSNF-000088 could be a good option to increase nicotinamide levels in plasma to achieve higher concentrations in the CNS target as reflected in the CSF without any obvious safety or drug interaction issues in mouse models [29].

## Conclusion

Our data indicate that high mean levels of nicotinamide can be achieved in blood. However, only a small subset of participants with early AD had measurable nicotinamide in CSF, the presumed site of action for CSF p-Tau231 lowering. In the small number of participants for whom nicotinamide reached measurable levels in the CSF, there appeared to be an associated reduction in CSF pTau_231_, but not other AD pTau or Aβ biomarkers. Overall, there are significant pharmaceutical and bioavailability limitations even with the high doses of oral nicotinamide used in the NEAT trial. The most critical problem with oral nicotinamide appears to be methyl metabolic inactivation. Inhibiting methylation may improve the bioavailability of nicotinamide. At present, these problems remain unsolved and limit its use in AD.

## Supplementary Information

Supplementary Material 1.

## Data Availability

Data from the NEAT trial is available from the ADCS Legacy project (https://www.adcs.org/data-sharing/).

## Abbreviations

NAD^+^/NADH: Nicotinamide Adenine Dinucleotide
HDAC: Histone Deacetylase
PI3k: Phosphoinositide 3-kinase
MAPK/ERK: Mitogen-Activated Protein kinase / Extracellular Signal-regulated kinase
cAMP: Cyclic adenosine 3,5-monophosphate
AD: Alzheimer’s Disease
Aβ: Amyloid-Beta
CNS: Central Nervous System
PO: Per oral
BID: Twice a day
TCA: Tricaboxylic Acid Cycle
PARP: Poly(ADP-ribose) polymerase
CD38: Cluster of Differentiation 38, cyclic ADP ribose hydrolase
NEAT: Nicotinamide as an Early AD Treatment
MCI: Mild Cognitive Impairment
CSF: Cerebrospinal Fluid
PK: Pharmacokinetics
PD: Pharmacodynamics
pTau: Phosphorylated Tau
LCMS: Liquid Chromatography Mass Spectrometry
IS: Internal Standard
QC: Quality Control
UPLC: Ultra Performance Liquid Chromatography
ESI^+^: Electrospray Positive Ion mode
CSH: Charged Surface Hybrid
V: Voltage
MNA: Methyl-nicotinamide
ApoE: Apolipoprotein E
Hr.: Hours
M6: Month 6
M12: Month 12
NNMT: Nicotinamide n-Methyltransferase
3xTg: Triple Transgenic mouse
IC_50_: Half-maximal Inhibitory Concentration
MR: Magnetic Resonance
SAM: S-adenosyl-l-methionine
Ki: Binding Affinity
