# Supplementary material for: Pharmacokinetic and pharmacodynamic assessment of oral nicotinamide in the NEAT clinical trial for early Alzheimer’s disease
Source: Alzheimers Res Ther. 2025 Mar 11;17:59. doi: 10.1186/s13195-025-01693-y (PMC11895359; doi:10.1186/s13195-025-01693-y)
Supplement: Supplementary file 1 — Supplementary Material 1. [file 13195_2025_1693_MOESM1_ESM.docx]

Supplementary mass spectrometry methods

Multiple reaction monitoring parameters (Supplementary **Table 1**) were optimized using the Waters MassLynx QuantOptimize Analyzer software (v4.1 SCN805). MassLynx 4.1 software was used to acquire, process, and analyze the data.

Supplementary Table 1. Mass spectrometry settings for optimum detection of the indicated analytes.

| **Analyte** | **Parent/Daughter m/z Transitions** | Dwell (sec) | Cone Voltage (V) | Collision energy (V) |
| --- | --- | --- | --- | --- |
| Nicotinamide | 123 > 80 | 0.2 | 10 | 25 |
| Nicotinic Acid | 124 > 80 | 0.2 | 10 | 30 |
| Nicotinamide-d4 | 127>84 | 0.2 | 10 | 25 |
| Nicotinamide mononucleotide | 335 > 123 | 0.2 | 10 | 20 |
| NAD^+^ | 664 >136 | 0.2 | 20 | 40 |
| Methyl-nicotinamide | 137 > 94 | 0.2 | 20 | 20 |

Precision and Specificity for Method Validation

Precision of the assay was determined by quantification from sample triplicates. For samples with measurable nicotinamide intraday covariance was 20% and methyl-nicotinamide 19%. 18% of patient samples with greater than 30% coefficient of variance were rerun with analyte CV down to 12 and 7%, respectively. Measurement of analyte recovery in biological matrix was calculated by dividing the measured area of deuterated nicotinamide in the sample by the corresponding value of deuterated nicotinamide in the external standard without matrix. Matrix effects reduced the internal standard to an average of 31% of its initial concentration. We found an R^2^ > 0.99 for a linear fit of responses to 7 three-fold dilutions in external standards of nicotinamide and methyl-nicotinamide beginning at 3333 nM. Accuracy of analyte spiked into plasma matrix was 116% of expected, after adjustment with nicotinamide-d4 internal standard.


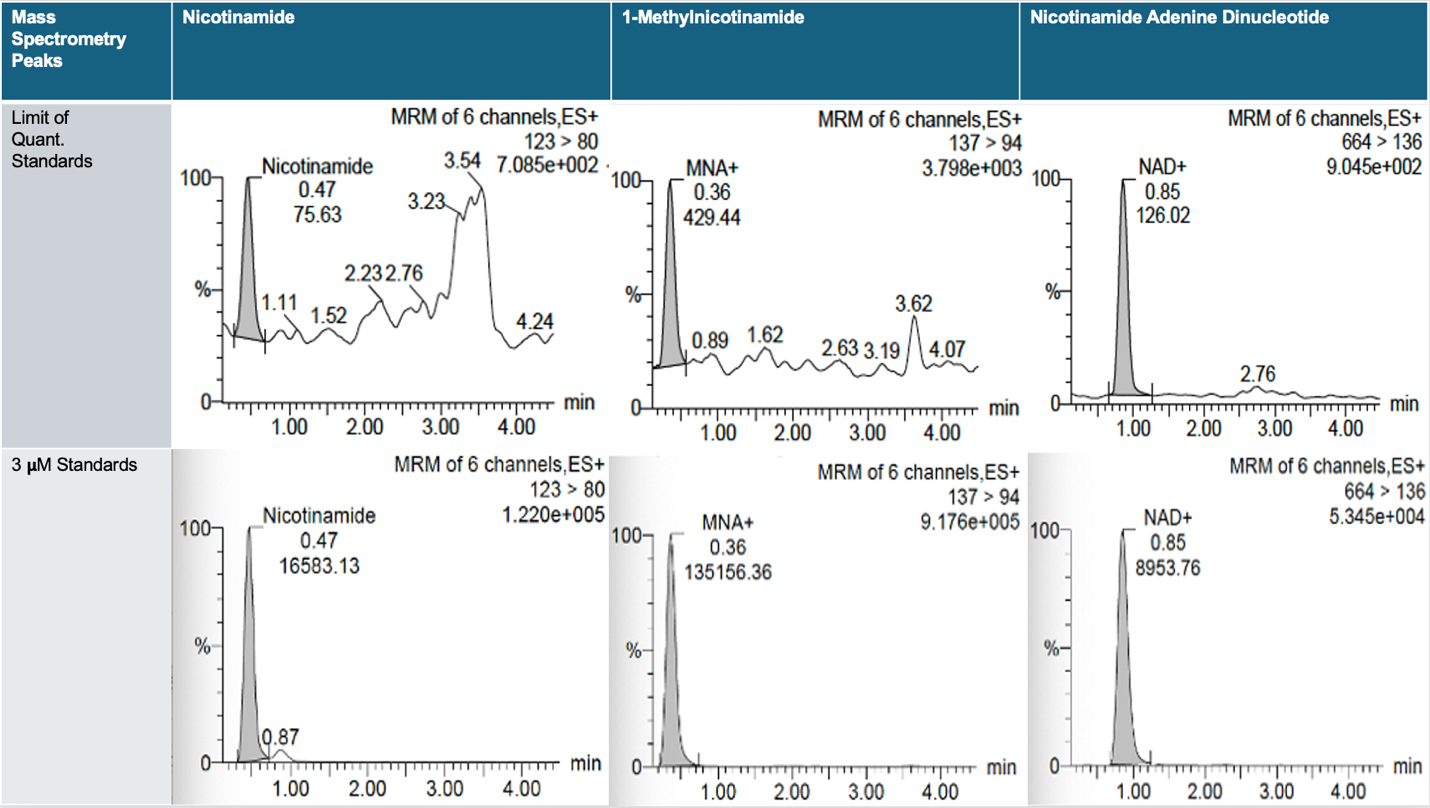


Supplementary Figure 1. Column chromatograms for nicotinamide and methyl-nicotinamide (MNA). Top row, at the limit of detection. Bottom row, in the 3 μM standard .


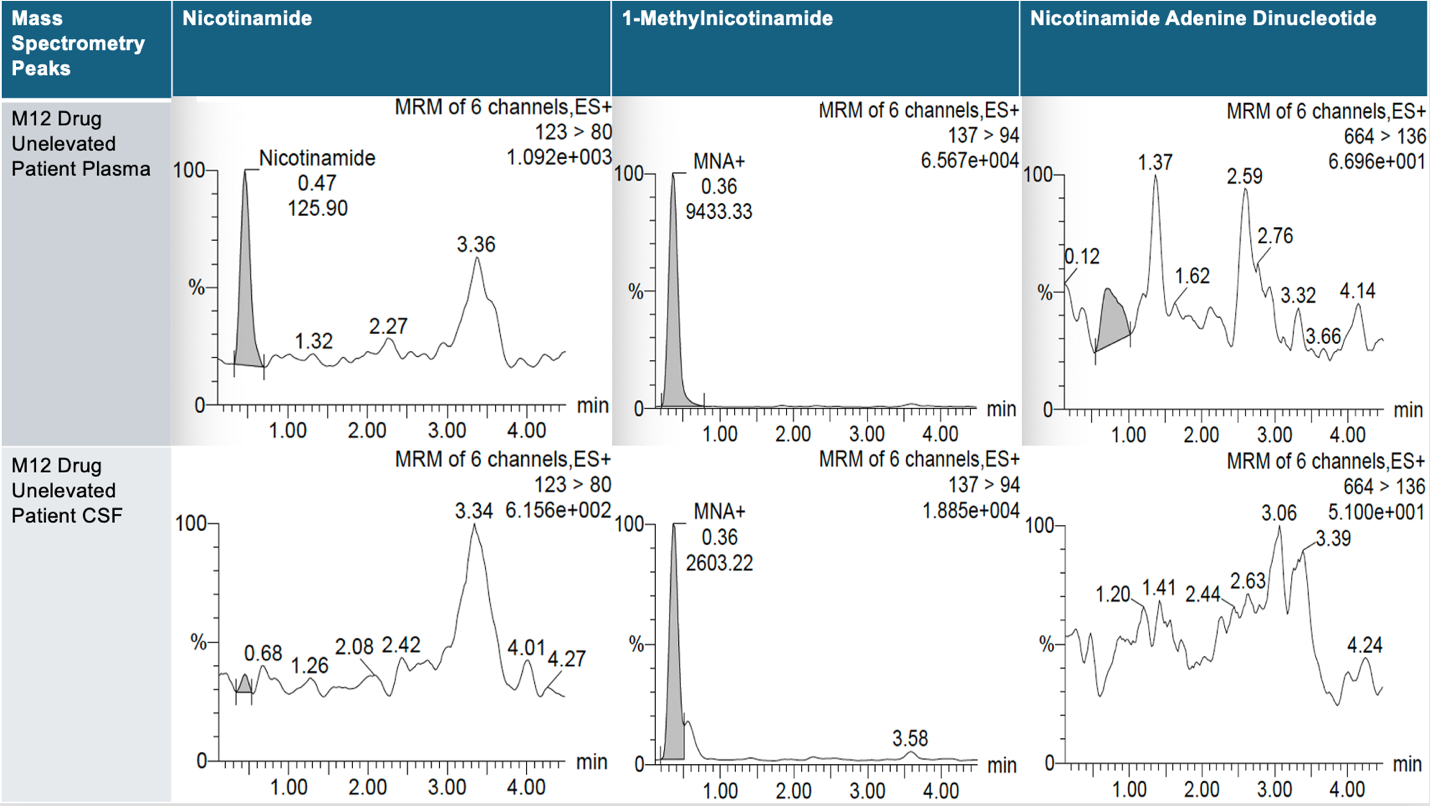


Supplementary Figure 2. Chromatograms for nicotinamide and methyl-nicotinamide (MNA) from a patient plasma sample at 12 months.

.

**Supplementary Figure 1** shows multi-reaction monitoring chromatograms specificity with sharp and symmetric analyte peak shapes, within 0.1 atomic mass units in 3 μM standards. **Supplementary** **Figure 2** shows chromatograms in patient plasma samples.

| Supplementary Table 2. Ketron NEAT PK PD Final Data 240715.xlsx | | | | | | Blanks had no sample. | | |  |  | Date Format: YY/MM/DD | | | |  | |  | |  | |  | |
| --- | --- | --- | --- | --- | --- | --- | --- | --- | --- | --- | --- | --- | --- | --- | --- | --- | --- | --- | --- | --- | --- | --- |
|  | Nicotinamide Limit of Quantification:  0.4 uM | | | | | Methylnicotinamide Limit of Quantification: 0.1 uM | | | | |  |  |  |  | |  | |  | |  | |  |
|  | Nicotinamide (uM) | | | | | 1-Methynicotinamide (uM) | | | | |  |  |  |  | |  | |  | |  | |  |
|  | Plasma | | | CSF | | Plasma | | | CSF | |  |  |  | PTAU231 | | | | | | | |  |
| NEAT Sample | baseline | Month 6 | Month 12 | baseline | Month 12 | baseline | Month 6 | Month 12 | baseline | Month 12 | Date of Run | TX | RID | baseline | | Month 12 | | change | | % change | |  |
| NEAT0290005 | 0.4 | 9.0 | 0.4 | 0.4 | 0.4 | 1.6 | 191.6 | 1.2 | 0.1 | 0.1 | 231115 | 1500mg | 5 | 49.5 | | 41 | | -8.5 | | -17 | |  |
| NEAT0290006 | 0.4 | 183.5 | 58.0 | 0.4 | 0.4 | 0.1 | 68.1 | 76.9 | 0.1 | 0.1 | 240208 | 1500mg | 6 | 7.7 | | 6.2 | | -1.5 | | -19 | |  |
| NEAT0350008 | 0.4 | 232.9 | 1.7 | 0.4 | 0.4 | 0.1 | 186.9 | 15.0 | 0.1 | 3.6 | 231115 | 1500mg | 8 | 42.3 | | 37 | | -5.3 | | -13 | |  |
| NEAT0290016 | 0.4 | 135.4 | 120.9 | 0.4 | 0.4 | 0.1 | 72.2 | 101.4 | 0.1 | 0.1 | 240208 | 1500mg | 16 | 46.3 | | 34.3 | | -12 | | -26 | |  |
| NEAT0290017 | 0.4 | 0.4 | 0.4 | 0.4 |  | 0.1 | 6.7 | 6.8 | 0.1 |  | 231204 | 1500mg | 17 | 29.3 | |  | |  | |  | |  |
| NEAT0350018 | 0.4 |  | 0.4 | 0.4 |  | 0.1 |  | 0.5 | 0.1 |  | 231115 | 1500mg | 18 | 67.8 | |  | |  | |  | |  |
| NEAT0290028 | 0.4 | 187.6 | 243.4 | 0.4 | 0.4 | 0.1 | 98.7 | 93.1 | 0.1 | 0.1 | 231204 | 1500mg | 28 | 74.8 | | 98.8 | | 24 | | 32 | |  |
| NEAT0290037 | 0.4 | 0.4 | 0.4 | 0.4 | 0.4 | 0.1 | 8.5 | 19.8 | 0.1 | 0.1 | 240208 | 1500mg | 37 | 86 | | 66 | | -20 | | -23 | |  |
| NEAT0290042 | 0.4 | 0.4 | 61.5 | 0.4 |  | 0.1 | 73.1 | 119.3 | 0.1 |  | 231204 | 1500mg | 42 | 10.5 | |  | |  | |  | |  |
| NEAT0290046 | 0.4 | 146.8 | 110.6 | 0.4 | 51.3 | 0.1 | 120.1 | 87.2 | 0.1 | 5.9 | 240208 | 1500mg | 46 | 55.8 | | 31.3 | | -24.5 | | -44 | |  |
| NEAT0290056 | 0.4 | 74.0 | 71.8 | 0.4 | 89.8 | 0.1 | 98.7 | 118.8 | 0.1 | 6.5 | 240208 | 1500mg | 56 | 15.1 | | 12.2 | | -2.9 | | -19 | |  |
| NEAT0290060 | 0.4 | 0.4 | 0.4 | 0.4 | 0.4 | 0.1 | 30.7 | 0.1 | 0.1 | 0.1 | 240116 | 1500mg | 60 | 25 | | 14.6 | | -10.4 | | -42 | |  |
| NEAT0350061 | 0.4 | 111.2 | 108.5 | 0.4 | 129.5 | 0.1 | 77.4 | 127.8 | 0.1 | 4.1 | 240126 | 1500mg | 61 | 32.8 | | 15.1 | | -17.7 | | -54 | |  |
| NEAT0350070 | 0.4 |  | 0.4 | 0.4 | 1.9 | 0.1 |  | 33.8 | 0.1 | 5.6 | 240126 | 1500mg | 70 | 39.3 | | 68.8 | | 29.5 | | 75 | |  |
| NEAT0290071 | 0.4 | 8.1 | 33.6 | 0.4 | 14.9 | 0.1 | 114.1 | 71.4 | 0.1 | 5.7 | 240208 | 1500mg | 71 | 32.8 | | 21.2 | | -11.6 | | -35 | |  |
| NEAT0290080 | 0.4 | 111.8 | 34.6 | 0.4 | 0.4 | 0.1 | 160.6 | 101.3 | 0.1 | 0.1 | 240208 | 1500mg | 80 | 7.8 | | 10.9 | | 3.1 | | 40 | |  |
| NEAT0290088 | 0.4 |  | 0.4 |  | 0.4 | 0.1 |  | 15.3 |  | 0.1 | 240116 | 1500mg | 88 | 21.4 | | 29 | | 7.6 | | 36 | |  |
| NEAT0290090 | 0.4 | 1.6 | 85.4 | 0.4 | 0.4 | 0.1 | 61.9 | 73.9 | 0.1 | 1.4 | 240208 | 1500mg | 90 | 48 | | 49.5 | | 1.5 | | 3 | |  |
| NEAT0290096 | 0.4 |  |  |  |  | 0.1 |  |  |  |  | 240116 | 1500mg | 96 | 10.1 | |  | |  | |  | |  |
| NEAT0290098 | 0.4 | 62.7 | 75.2 | 0.4 | 0.4 | 0.1 | 130.4 | 116.0 | 0.1 | 1.3 | 240116 | 1500mg | 98 | 6.9 | | 6.6 | | -0.3 | | -4 | |  |
| NEAT0290100 | 0.4 | 20.2 | 70.7 | 0.4 | 13.1 | 0.1 | 151.9 | 293.9 | 0.1 | 6.4 | 240116 | 1500mg | 100 | 67.5 | | 52.5 | | -15 | | -22 | |  |
| NEAT0290106 | 0.4 | 150.5 | 123.7 | 0.4 |  | 0.1 | 186.7 | 219.6 | 0.1 |  | 240208 | 1500mg | 106 | 32.5 | |  | |  | |  | |  |
| NEAT0290108 | 0.4 | 0.4 | 2.1 | 0.4 | 0.4 | 0.1 | 248.2 | 242.1 | 0.1 | 7.5 | 240126 | 1500mg | 108 | 20.4 | | 20.1 | | -0.3 | | -1 | |  |
| NEAT0290109 | 0.4 | 26.4 | 2.5 | 0.4 | 44.8 | 0.1 | 186.6 | 152.9 | 0.1 | 9.2 | 240126 | 1500mg | 109 | 90.5 | | 65.3 | | -25.2 | | -28 | |  |
| NEAT0290003 | 0.4 | 0.4 | 0.40 | 0.4 | 0.4 | 0.9 | 0.1 | 0.1 | 0.1 | 0.1 | 231115 | PLACEBO | 3 | 66.3 | | 44 | | -22.3 | | -34 | |  |
| NEAT0290009 | 0.4 | 0.4 | 0.40 | 0.4 | 0.4 | 0.3 | 0.3 | 0.4 | 0.1 | 0.1 | 231204 | PLACEBO | 9 | 32.5 | | 26.5 | | -6 | | -18 | |  |
| NEAT0290012 | 0.4 | 0.4 | 0.40 | 0.4 | 0.4 | 0.1 | 0.1 | 0.1 | 0.1 | 0.1 | 231204 | PLACEBO | 12 | 23.6 | | 22.6 | | -1 | | -4 | |  |
| NEAT0350019 | 0.4 | 0.4 | 0.40 | 0.4 | 0.4 | 0.1 | 0.1 | 0.1 | 0.1 | 0.1 | 240126 | PLACEBO | 19 | 22.7 | | 15.9 | | -6.8 | | -30 | |  |
| NEAT0290024 | 0.4 | 0.4 | 0.40 | 0.4 | 0.4 | 0.1 | 0.1 | 0.1 | 0.1 | 0.1 | 231204 | PLACEBO | 24 | 3.6 | | 3.7 | | 0.1 | | 3 | |  |
| NEAT0290025 | 0.4 | 0.4 | 0.40 | 0.4 | 0.4 | 0.1 | 0.1 | 0.1 | 0.1 | 0.1 | 240208 | PLACEBO | 25 | 57 | | 48 | | -9 | | -16 | |  |
| NEAT0350030 | 0.4 | 0.4 | 0.40 | 0.4 | 0.4 | 0.1 | 0.1 | 0.1 | 0.1 | 0.1 | 240126 | PLACEBO | 30 | 81 | | 104.8 | | 23.8 | | 29 | |  |
| NEAT0290043 | 1.6 | 0.4 | 0.40 | 0.4 | 0.4 | 0.1 | 0.1 | 0.1 | 0.1 | 0.1 | 231204 | PLACEBO | 43 | 66 | | 49 | | -17 | | -26 | |  |
| NEAT0350044 | 0.4 | 0.4 | 0.40 | 0.4 | 0.4 | 0.1 | 0.1 | 0.1 | 0.1 | 0.1 | 240126 | PLACEBO | 44 | 48.8 | | 55 | | 6.2 | | 13 | |  |
| NEAT0290049 | 0.4 | 0.4 | 0.40 | 0.4 | 0.4 | 0.1 | 0.1 | 0.1 | 0.1 | 0.1 | 240208 | PLACEBO | 49 | 61 | | 52.3 | | -8.7 | | -14 | |  |
| NEAT0290051 | 0.4 |  |  |  |  | 0.1 |  |  |  |  | 231204 | PLACEBO | 51 | 60.5 | |  | |  | |  | |  |
| NEAT0290057 | 0.4 | 0.4 | 0.40 | 0.4 | 0.4 | 0.1 | 0.1 | 0.1 | 0.1 | 0.1 | 231204 | PLACEBO | 57 | 25.8 | | 23.4 | | -2.4 | | -9 | |  |
| NEAT0350059 | 0.4 |  | 0.40 | 0.4 | 0.4 | 0.1 |  | 0.1 | 0.1 | 0.1 | 240126 | PLACEBO | 59 | 59 | | 44.3 | |  | |  | |  |
| NEAT0290062 | 0.4 |  |  |  |  | 0.1 |  |  |  |  | 240116 | PLACEBO | 62 | 29.8 | |  | |  | |  | |  |
| NEAT0290067 | 0.4 | 0.4 | 0.40 | 0.4 | 0.4 | 0.1 | 0.1 | 0.1 | 0.1 | 0.1 | 240116 | PLACEBO | 67 | 35.3 | | 38.3 | | 3 | | 8 | |  |
| NEAT0290083 | 0.4 | 0.4 | 0.40 | 0.4 | 0.4 | 0.1 | 0.1 | 0.8 | 0.1 | 0.1 | 240116 | PLACEBO | 83 | 23 | | 25.3 | | 2.3 | | 10 | |  |
| NEAT0290084 | 0.4 | 0.4 | 0.40 | 0.4 | 0.4 | 0.1 | 0.1 | 0.1 | 0.1 | 0.1 | 240116 | PLACEBO | 84 | 64.8 | | 54.8 | | -10 | | -15 | |  |
| NEAT0290091 | 0.4 | 0.4 | 0.40 | 0.4 | 0.4 | 0.1 | 0.1 | 0.1 | 0.1 | 0.1 | 240116 | PLACEBO | 91 | 16.7 | | 20.3 | | 3.6 | | 22 | |  |
| NEAT0290092 | 0.4 | 0.4 | 0.40 | 0.4 | 0.4 | 0.1 | 0.1 | 0.1 | 0.1 | 0.1 | 240116 | PLACEBO | 92 | 53.8 | | 47.8 | | -6 | | -11 | |  |
| NEAT0290093 | 0.4 | 0.4 | 0.40 | 0.4 | 0.4 | 0.1 | 0.1 | 0.1 | 0.1 | 0.1 | 240116 | PLACEBO | 93 | 27.5 | | 40.3 | | 12.8 | | 47 | |  |
| NEAT0290099 | 0.4 | 0.4 | 0.40 | 0.4 | 0.4 | 0.1 | 0.1 | 0.1 | 0.1 | 0.1 | 240116 | PLACEBO | 99 | 6.9 | | 6.7 | | -0.2 | | -3 | |  |
| NEAT0290101 | 0.4 |  |  |  |  | 0.1 |  |  |  |  | 240126 | PLACEBO | 101 | 3.5 | |  | |  | |  | |  |
| NEAT0290102 | 0.4 | 0.4 | 0.40 | 0.4 | 0.4 | 0.1 | 0.1 | 0.1 | 0.1 | 0.1 | 240126 | PLACEBO | 102 | 3.4 | | 3.6 | | 0.2 | | 6 | |  |
